# NAP (Davunetide): The Neuroprotective ADNP Drug Candidate Penetrates Cell Nuclei Explaining Pleiotropic Mechanisms

**DOI:** 10.3390/cells12182251

**Published:** 2023-09-11

**Authors:** Maram Ganaiem, Nina D. Gildor, Shula Shazman, Gidon Karmon, Yanina Ivashko-Pachima, Illana Gozes

**Affiliations:** 1 The Elton Laboratory for Molecular Neuroendocrinology, Department of Human Molecular Genetics and Biochemistry, Faculty of Medicine, Sagol School of Neuroscience and Adams Super Center for Brain Studies, Tel Aviv University, Tel Aviv 6997801, Israel; maramg@mail.tau.ac.il (M.G.); ngildor@gmail.com (N.D.G.); gidikarmon@gmail.com (G.K.); yaninaiv11@gmail.com (Y.I.-P.); 2 Department of Mathematics and Computer Science, The Open University of Israel, Raanana 4353107, Israel; shula.shazman@openu.ac.il; 3 Department of Information Systems, The Max Stern Yezreel Valley College, Yezreel Valley, Afula 1930600, Israel

**Keywords:** activity-dependent neuroprotective protein (ADNP), ADNP syndrome, CRISPR/Cas9, green fluorescent protein (GFP), immunocytochemistry, live cell imaging, reverse transcription polymerase chain reaction (RT-PCR), ketamine, NAP (davunetide)

## Abstract

(1) Background: Recently, we showed aberrant nuclear/cytoplasmic boundaries/activity-dependent neuroprotective protein (ADNP) distribution in ADNP-mutated cells. This malformation was corrected upon neuronal differentiation by the ADNP-derived fragment drug candidate NAP (davunetide). Here, we investigated the mechanism of NAP nuclear protection. (2) Methods: CRISPR/Cas9 DNA-editing established N1E-115 neuroblastoma cell lines that express two different green fluorescent proteins (GFPs)—labeled mutated ADNP variants (p.Tyr718* and p.Ser403*). Cells were exposed to NAP conjugated to Cy5, followed by live imaging. Cells were further characterized using quantitative morphology/immunocytochemistry/RNA and protein quantifications. (3) Results: NAP rapidly distributed in the cytoplasm and was also seen in the nucleus. Furthermore, reduced microtubule content was observed in the ADNP-mutated cell lines. In parallel, disrupting microtubules by zinc or nocodazole intoxication mimicked ADNP mutation phenotypes and resulted in aberrant nuclear–cytoplasmic boundaries, which were rapidly corrected by NAP treatment. No NAP effects were noted on ADNP levels. Ketamine, used as a control, was ineffective, but both NAP and ketamine exhibited direct interactions with ADNP, as observed via in silico docking. (4) Conclusions: Through a microtubule-linked mechanism, NAP rapidly localized to the cytoplasmic and nuclear compartments, ameliorating mutated ADNP-related deficiencies. These novel findings explain previously published gene expression results and broaden NAP (davunetide) utilization in research and clinical development.

## 1. Introduction

First identified in our laboratory, activity-dependent neuroprotective protein (ADNP) [1] is vital for brain formation [2]. ADNP encompasses many active domains [3,4] and is extremely conserved between humans and mice (90% identity at the mRNA level) [5]. Our original sequencing identified an ADNP homeobox domain profile (within the 1102 amino acids in human ADNP), which is known to be associated with cell morphogenesis and differentiation [5] and is now suggested to be associated with DNA repair [6]. We also uncovered the nuclear localization and transcription factor activities of ADNP, as it regulates the chromatin structure and is implicated in the expression of hundreds of essential genes [4,7,8,9,10,11,12]. These findings have been substantiated and extended by others [6,13,14,15,16,17,18,19]. Most recent clinical findings include the association of de novo ADNP mutations in congenital cranial anomalies [20].

ADNP syndrome (also known as Helsmoortel-Van Der Aa syndrome) is a rare condition characterized by autistic/intellectual disabilities, such as memory and communication problems and prominent sensory seeking and muscle weakness, accompanied by brain pathology and tauopathy [12,21,22,23,24,25,26,27,28]. The syndrome occurs when one allele of the *ADNP* gene undergoes a de novo mutation, resulting in the loss of normal functions [10] and expressing distinct mutation-specific epigenetic signatures [29,30], which may also be linked with differential phenotypic outcomes [31]. Furthermore, we revealed an accumulation of somatic mutations in the *ADNP* gene in post-mortem Alzheimer’s disease brains and correlated this accumulation with the progression of tauopathy [32].

When analyzing the ADNP sequence, we revealed a short neuroprotective peptide sequence, NAPVSIPQ (NAP, corresponding to the drug candidate davunetide, also known as AL-108 or CP201) [1]. We then identified NAP as a microtubule/cytoskeleton-interacting neuroprotective domain [3,33] that directly binds through its SIP motif with microtubule end-binding proteins EB1 and EB3 [33]. ADNP deficiencies and *ADNP* gene mutations in transgenic mice cause dramatic alterations in mRNA/protein expression profiles [8,9,34,35] as well as slower microtubule-dependent axonal transport [9], aberrant dendritic spine formation [10,35], and tauopathy [10,34]. NAP replaces/repairs these deficiencies by working as a crucial ADNP fragment that enhances microtubule dynamics and Tau–microtubule interactions [36], protecting against Tau hyper-phosphorylation [34,37] and tauopathy [10,34], regulating actin–cytoskeletal association [3], and linking with WNT signaling [16,38] to lead to dendritic spine formation [10,33,35], which translates to normalized development and behavioral protection [34,35,39].

More specifically, to directly address the corrective effects of NAP (10^−12^ M, 4 h) on ADNP mutations, we have resorted to neuronal-like cells transiently transfected with human ADNP mutated plasmids. As such, neuronal-like N1E-115 mouse neuroblastoma cells were co-transfected with plasmids expressing EB3-red fluorescence protein (RFP) and green fluorescence protein (GFP)–ADNP or GFP-mutated-ADNP and subjected to live cell imaging. Two parameters of EB3 mobility were used to assess microtubule dynamics, namely the growth track length and growth track speed of EB3 comet-like structures, reflecting the lengths of the microtubule growing events and the speed of microtubule assembly, respectively [36]. In these tests, we revealed that the overexpression of full-length ADNP significantly increased EB3 comet track length and that NAP treatment did not further influence this increase. Comparing the effects of ADNP p.Arg730*, p.Tyr719* [32] p.Glu830synfs*83, or p.Ser404* [3] indicated that each of these tested mutations significantly decreased EB3 comet speeds and track lengths compared to full-length ADNP. NAP treatment significantly augmented the growth speed and track length of the EB3 comets with all mutations [3,32]. When further considering EB3 comet speed, reductions by both ADNP p.Glu830synfs*83 and p.Ser404* were noted and corrected by NAP treatment, with ADNP p.Ser404* having the most impact and NAP providing significant protection [3]. Together, these previous results indicate ADNP’s regulation of microtubule dynamics as well as the subtle differential effects of various ADNP mutations and NAP’s corrective effects.

To extend these findings, we employed CRISPR/Cas9 genome editing in the above cellular model, namely N1E-115 mouse neuroblastoma cells, to form neuron-like cell lines constitutively expressing ADNP mutant proteins conjugated to GFP. These new cell lines were characterized by using quantitative morphology, immunocytochemistry, and live cell imaging. These novel cell lines constitutively expressing GFP-ADNP p.Pro403 (p.Ser404* human orthologue, above) and GFP-ADNP p.Tyr718* (p.Tyr719* human orthologue, above) revealed new and distinct phenotypes. We discovered increased neurite numbers in the neuron-like cells expressing the p.Pro403* mutant and decreased survival of cells expressing the p.Tyr718* variant. These results were associated with an increased cytoplasmatic expression of both mutant proteins (as verified via tubulin and DAPI staining). Furthermore, reduced nuclear/cytoplasmic boundaries were observed in both mutant cell lines but were dramatically accentuated in the GFP-ADNP p.Tyr718* cell line; these mutant-induced undiscernible nuclear envelopes were then corrected by NAP. Thus, we showed that different ADNP mutants can be characterized by distinct phenotypes and reveal aberrant cytoplasmic–nuclear crosstalk [40].

Here, aiming to expand our knowledge regarding the potential nuclear activity of NAP, we further examined the mechanism by which NAP corrects the adverse effect of truncated ADNP forms in the cell. Cells were incubated for 7 days with standard medium, and then NAP-conjugated to Cy5, which was added immediately before confocal live imaging. Our results indicated NAP presence in and around the nucleus of the full-length ADNP conjugated to the GFP cells and of both mutated (truncated) ADNP cell lines. Furthermore, NAP provided immediate protection against indiscernible nuclear envelope/mutated GFP-ADNP cytoplasmic–nuclear distribution.

We then hypothesized that NAP protection against indiscernible nuclear envelope/mutated GFP-ADNP cytoplasmic–nuclear distribution was mediated via microtubule protection, an ADNP/NAP neuroprotective target. Using zinc, a microtubule disrupting agent [36], and nocodazole [41], we tested whether microtubule disassembly causes similar ADNP cytoplasmic–nuclear aberrant distribution through mimicking the phenotype of ADNP-mutated neuronal-like cells. Our results showed that both the zinc- and the nocodazole-treated cells exhibited a diffused ADNP pattern that was restored via NAP treatment.

As a control, we used low-dose ketamine (previously suggested to increase ADNP content) [42]. Our results showed that, in the current experimental system, neither NAP nor ketamine increased *Adnp* mRNA content. Surprisingly, using in silico docking, we discovered ketamine–ADNP–EB1–EB3 interactions, partially explaining NAP protection against ketamine intoxication [43].

In short, we discovered that NAP rapidly entered the cell cytoplasm and nucleus and immediately corrected mutant-induced indiscernible cytoplasmic–nuclear distribution through microtubule protection.

## 2. Materials and Methods

### 2.1. Cell Culture and Treatments

As previously described [36,40], mouse neuroblastoma N1E-115 cell clones (ATCC, catalogue number, CRL-2263™, Bethesda, MD, USA) were maintained and differentiated into neuronal-like cells as before, i.e., incubated in reduced fetal bovine serum (2%) and DMSO (1.25%)-containing medium for seven days [40,44].

### 2.2. CRISPR/Cas9 DNA-Editing System

CRISPR/Cas9 genome editing was used to develop ADNP-mutated cell lines as recently described [40].

### 2.3. Antibodies

Mouse monoclonal anti-tubulin antibodies (TUB2.1), a generous gift from Professor Colin J. Barnstable [45], were diluted 1:100 for immunocytochemistry. The secondary antibodies used for immunocytochemistry were goat anti-mouse Alexa Fluor 488-conjugated antibodies (diluted 1:500) (Invitrogen by Thermo Fisher Scientific, Eugene, OR, USA). Mouse monoclonal beta-actin antibody was diluted 1:1000 for immunoblotting (Sc-517582, Santa Cruz Biotechnology Dallas, TX, USA). GFP antibodies were used for Western analysis as before [40].

### 2.4. Immunocytochemistry

Cells were plated on 24-well plates at a concentration of 25 × 10^4^ cells per well. After 7 days with differentiated medium, cells were immunostained with mouse monoclonal tubulin antibodies (TUB2.1) [45], followed by incubation with goat anti-mouse Alexa Fluor 488-conjugated antibodies, as previously performed [40]. Confocal microscopy was carried out as described in the live cell imaging section below. The tubulin intensity in the cells was measured using ImageJ Fiji software (2.9.0, 2022) (National Institutes of Health, Bethesda, MD, USA) [46]. Overall, 55–100 cells (1024 × 1024 pixels/image) were analyzed.

### 2.5. Live Cell Imaging

Cells were plated on 35 mm dishes (#81156, 60 μ-Dish, Ibidi, Martinsried, Germany) at a concentration of 25 × 10^4^ cells per dish. After 7 days in culture, (Cy5) **NAP** (1 nM) (generously provided by Dr. Doron Chabat) [36] was added immediately before confocal microscopy (objective × 100 (PL Apo) oil immersion, NA 1.4, pinhole size 3 with argon laser at wavelength of 496 nm, Leica TCS SP8, Leica Microsystems, Wetzlar, Germany) visualization [36]. Using ImageJ Fiji software [46], we measured (Cy5) **NAP** intensity in and around the nuclei (about 100 cells were visualized and quantitated). Moreover, the diffusely localized ADNP in the cells was measured as before [40].

A separate parallel control experiment was carried out with ketamine (100 µM) *(*Vetoquinol, Lure, France). Here, 17 fields to each condition were analyzed, with an average of 13–20 cells in each field. The percentage of diffusely localized ADNP in cells was calculated in each field, as above and before [40].

### 2.6. Cellular Fractionations

Cells were plated on 10 cm dishes at a concentration of 1× 10^6^ cells per dish. Regarding cellular fractionations, to obtain crude nuclear fractions of cells exposed to (Cy5) **NAP** (1 nM) for twenty minutes, we used a centrifugation method as detailed in [47]. The nuclear fractions derived from two independent plates were suspended in fractionation buffer [HEPES 20 mM, KCL 10 mM, MgCl_2_ 2 mM, EDTA 1 mM, EGTA 1 mM, and DTT with protease inhibitory (PI) Cocktail that were added just before use]. The nuclear fractions were analyzed in duplicates using spectramaxELISA (Molecular Devices, San Jose, CA, USA) at a wavelength of 640 nm.

### 2.7. Microtubule Intoxication

To disrupt microtubule function, cells were treated for 4 h with zinc (400 µM) [36] after 7 days in culture with a differentiation medium. Sham-treated cells expressing full-length ADNP were compared to cells treated with zinc (400 µM) or zinc + NAP (1 nM) using live cell imaging [40]. Thirty-five images (1024 × 1024 pixels/image) were analyzed, and the percentage of diffusely localized ADNP outside the nucleus (aberrant nuclear boundaries) was calculated. Microtubule disruption was further achieved via treatment with nocodazole (10 µM) for 6 h; the cells were then washed with PBSx1 and treated with NAP (1 nM) for 1 h [41]. The microtubule stabilizer paclitaxel (5 µM, 2 h) [48] was used as a control alone or in combination with + NAP (1 nM). These different treatment groups were compared via live cell imaging as described above.

### 2.8. RNA Extraction and Quantitative Real-Time PCR

Neuronal-like cells were incubated with NAP (1 pM) [3] or ketamine (100 µM) for 4 h [42]. RNA extraction was performed at the end of the treatment period. RNA extraction (TRI Reagent, T9424, Sigma-Aldrich, Rehovot, Israel) and gene expression analysis were carried out as described before [35,49]. RNA expression levels were determined using the following specific mouse primers: Adnp sense 5′-ACGAAAAATCAGGACTATCGG-3′, antisense 5′-GGACATTCCGGAAATGACTTT-3′, GFP-Adnp 5′-CAACGAGAAGCGCGATCA-3′, antisense 5′-GGCCGCGTACAGCTCGT-3′. mRNA levels were normalized to Hprt sense 5′-GGATTTGAATCACGTTTGTGTC-3′ and anti-sense 5′-AACTTGCGCTCATCTTAGGC-3′, and GFP-Adnp transcripts were further normalized to Adnp transcripts. The expression results are presented as 2^−ΔCT^ [50,51].

### 2.9. Western Analysis

Western analysis was conducted to quantify microtubule bound and free tubulin and was performed as before [52] using TUB2.1 and actin antibodies (above). Western analysis with GFP-recognizing antibodies and actin-recognizing antibodies as a loading standard was also performed as before [40] on ketamine-treated mutated cell clones as above.

### 2.10. Experimental Timeline

**Figure 1:**
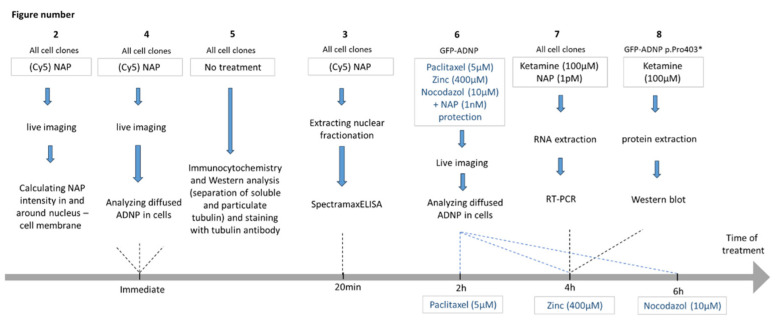
Experimental design. The figure numbers refer to the figures in the results. The experiments are described in broad terms in accordance with the optimal concentrations and timelines chosen based on previously published research (as cited in the text).

A graphical illustration of the experimental design is depicted below (Figure 1).

### 2.11. In Silico Modeling

I-TASSER [53] was used for protein structure modeling, and HDOCK [54] was used for the in silico protein/protein docking of ADNP, 1102 amino acids [5], microtubule end-binding proteins 1 and 3 (EB1 and EB3), and ADNP-external NAP (davuentide) interactions. HDOCK (as above) and SwissDock [55] were used for ketamine (S-Ketamine taken from pdb structure 7 sac). PyMOL(TM) version 2.5.2 (Schrodinger, Manheim, Germany) was used to create figures.

### 2.12. Statistical Analysis

Data are presented as the mean ± S.E.M. from at least two/three independent experiments. Statistical analysis of the data was performed via one-/two-way ANOVA (followed by Tukey’s post hoc test) using PRISM Statistics software, version 24 (IBM, Armonk, NY, USA); * *p* < 0.05, ** *p* < 0.01, *** *p* < 0.001.

## 3. Results

### 3.1. Exogenously Added NAP Is Present in and around the Nucleus

**Figure 2:**
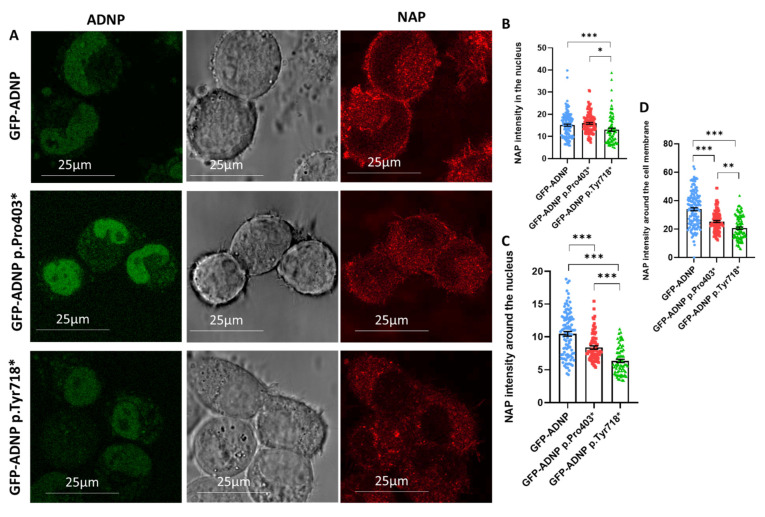
Live cell imaging of neuronal-like cells treated with (Cy5) **NAP** indicates NAP presence in and around the nucleus. (**A**) Representative confocal/fluorescent images after 7 days in culture involving full-length ADNP or truncated ADNP proteins conjugated to GFP (green); (Cy5) **NAP** was added immediately before microscopy (red). (**B**) Quantitative analysis of (Cy5) **NAP** intensity in the nucleus (~100 cells were analyzed) showed the presence of (Cy5) **NAP** in the nucleus [F _2, 251_ = 47.09]. (**C**,**D**) Quantitative analysis of (Cy5) **NAP** intensity around the nucleus (**C**) or cell membrane (**D**) (~100 cells were analyzed) indicated that (Cy5) **NAP** presence in and around the nucleus and cell membrane has a lower intensity in the ADNP p.Tyr718* mutation, the more severe ADNP mutation form [F_2, 333_ = 6.736]. * *p* < 0.05; ** *p* < 0.01; *** *p* < 0.001.

Figure 2A represents live cell images of GFP-ADNP (full-form of ADNP, used as a control), GFP-ADNP p.Pro403*, and GFP-ADNP p.Tyr718* expression immediately after the addition of (Cy5) **NAP** (namely, NAP labeled by Cy5). GFP-ADNP p.Pro403* was observed to be slightly more fluorescent. Concentrating on (Cy5) **NAP** showed distribution not only in the cytoplasm but also in the nucleus (Figure 2A, images; Figure 2B, quantifications). A lower intensity of (Cy5) **NAP** was observed in the GFP-ADNP p.Tyr718* cell clone (Figure 2B), which represents a more severe phenotype, with GFP-ADNP p.Pro403* increasing neurite numbers in the neuron-like cells, while the p.Tyr718* variant decreased the survival of cells [22,40]. Furthermore, both mutated cell lines showed the lower fluorescence intensity of (Cy5) **NAP** around the nuclear area (Figure 2C) and around the cell membrane (Figure 2D).

**Figure 3:**
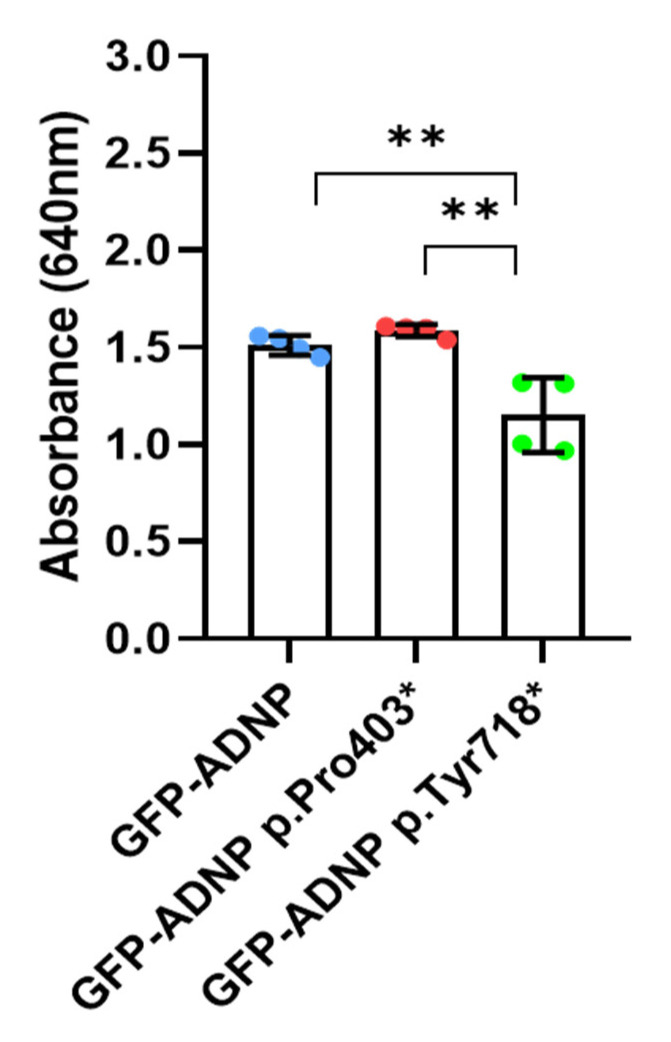
Spectrophotometric measurements of (Cy5) **NAP** indicate NAP presence in the nuclear fraction. Experiments were performed as detailed in Section 2. Nuclear preparations are shown; ** *p* < 0.01. Absorbance was measured using SpectramaxELISA.

To verify our confocal microscopy results, we utilized a cell fractionation protocol and assessed (Cy5) **NAP** presence in the nuclear fraction. Figure 3 shows significant (Cy5) **NAP** presence in the nuclear fraction of the ADNP p.Pro403* cells and reduced amounts in the cells carrying the ADNP p.Tyr718* mutation, both of which are in agreement with the confocal microscopy results (Figure 2).

### 3.2. Immediate Correction of Mutant-Induced Undiscernible Nuclear Envelopes by NAP

**Figure 4:**
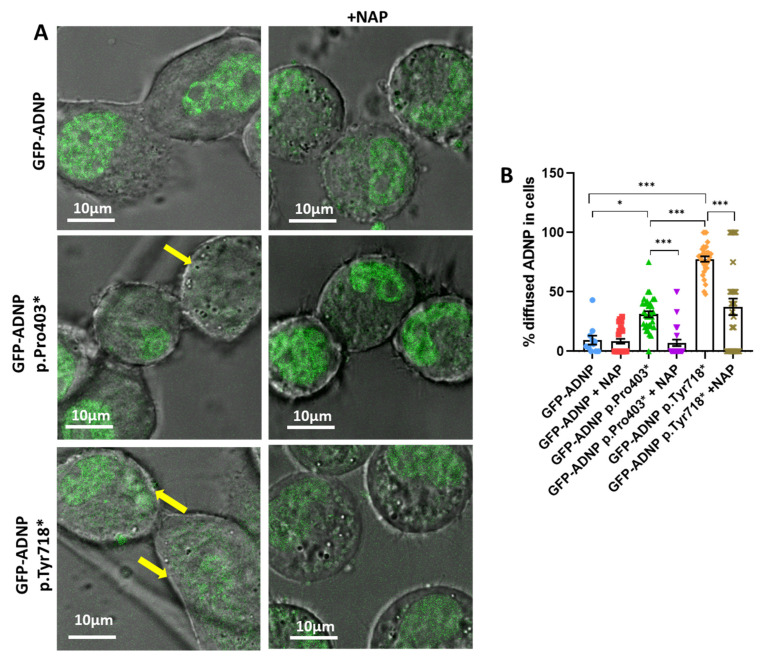
Live cell imaging with (Cy5) **NAP** treatment reveals an immediate correction of aberrant nuclear\cytoplasmic ADNP distribution. (**A**) Representative confocal-/fluorescent-merged images (Leica SP8) after 7 days in culture of neuroblastoma cells expressing full-length ADNP or truncated ADNP conjugated to GFP before and immediately after (Cy5) **NAP** was added. Selected cells showing diffusely localized GFP-ADNP p.Tyr718* are labeled with yellow arrows, indicating cells with indiscernible nuclear/cytoplasmic boundaries. (**B**) Comparisons between neuroblastoma cells expressing full-length ADNP or truncated ADNP conjugated to GFP after 7 days in culture and incubated with (Cy5) **NAP** (also including previously published controls) [40]. * *p* < 0.05; *** *p* < 0.001.

Here, we analyzed diffusely localized ADNP in cells expressing GFP-ADNP, GFP-ADNP p.Pro403*, and GFP-ADNP p.Tyr718* after (Cy5) NAP treatment (Figure 4A). Results showed that NAP treatment immediately corrected the aberrant cytoplasmic–nuclear distribution of mutated forms of ADNP (Figure 4A, confocal images without and with NAP; Figure 4B, quantification) [40]. The results indicated deleterious mutation effects (accentuated in the GFP-ADNP p.Tyr718* mutant) that were immediately ameliorated via (Cy5) **NAP** addition (as measured by the percentage of diffusely localized mutated ADNP in the cells) [F_5, 154_ = 55.97].

### 3.3. Tubulin/Microtubules Decrease as a Consequence of ADNP Mutations

**Figure 5:**
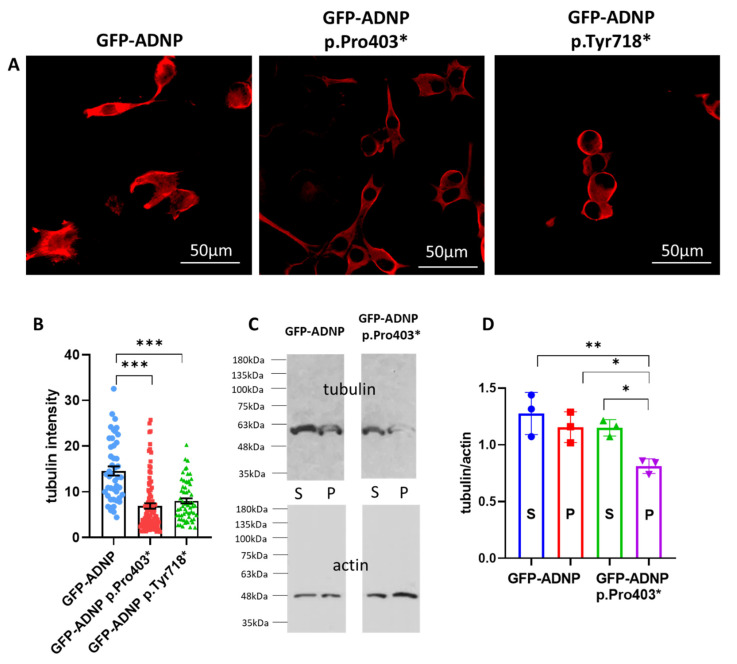
Immunostaining and Western blotting with tubulin antibody indicated a reduction in tubulin\microtubule staining in mutated ADNP cells. (**A**) Differentiated neuroblastoma neuronal-like cells expressing full-length ADNP or truncated ADNP were immunostained with tubulin antibodies (red). (**B**) Quantitative analysis of tubulin intensity in cells (55–100 cells) indicated lower microtubule intensity in cells expressing truncated ADNP [F_2, 162_ = 6.611], *** *p* < 0.001. (**C**) Western analysis of soluble (S) and particulate (P—microtubule bound) immunoreactive tubulin. (**D**) Densitometric scanning of two biological repeats, including one technical repeat [F_3, 8_ = 7.56]. * *p* < 0.05, ** *p* < 0.01.

As microtubules are a major target of NAP/ADNP [56] and microtubules are key to nuclear membrane integrity [57,58], we chose to investigate whether our clonal ADNP-mutated cells present tubulin\microtubules reductions, which impact nuclear/cytoplasmic ADNP-indiscernible organization. Using previously prepared tubulin immunostaining data [40] and measuring tubulin staining intensity in the neuronal-like cell lines indeed indicated a significant microtubule reduction (Figure 5A). The quantifying tubulin staining intensity revealed a highly significant decrease in tubulin\microtubule expression in the ADNP-mutated cell lines (Figure 5B). To further extract the soluble and microtubule-associated tubulin, separation via SDS polyacrylamide gel electrophoresis and tubulin quantitation via Western analysis (with the same antibody used for immunocytochemistry) revealed a similar reduction (using the clone GFP-ADNP p.Pro403 as an example, Figure 5C,D).

### 3.4. Zinc- and Nocodazole-Induced Microtubule Dysfunction Enhances Cytoplasmic/Nuclear Aberrant Morphology, Which Is Corrected by NAP Treatment

**Figure 6:**
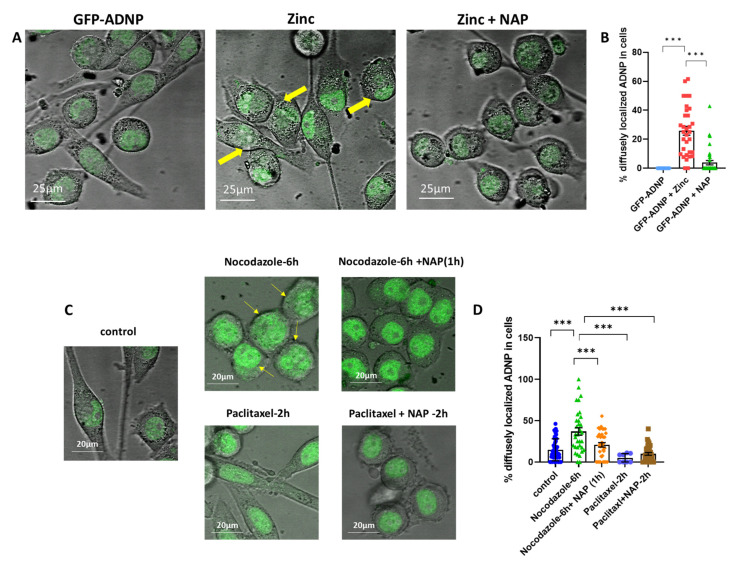
Zinc and nocodazole, which reduce microtubules, were shown to affect cytoplasmic/nuclear aberrant boundaries; however, this was corrected via NAP treatment. (**A**) Representative confocal/fluorescent-merged images after 7 days in culture with differentiation medium of control cells expressing full-length ADNP with and w/o 4 h zinc (400 µM) treatment or full-length ADNP with zinc (400 µM) + NAP (1 nM). (**B**) Thirty-five images were analyzed, and the percentage of diffusely localized ADNP outside the nucleus (aberrant nuclear boundaries) was calculated. Yellow arrows indicate indiscernible nuclear/cytoplasmic nuclear boundaries (as reflected via ADNP fluorescence) [F_2, 95_ = 49.15]; *** *p* < 0.001. (**C**,**D**) Similar to (**A**,**B**), only with nocodazole and paclitaxel instead of zine [F_4, 150_ = 14.46]; *** *p* < 0.001 (please see Figure 1 for more experimental details).

Further, we examined whether the aberrant nuclear envelope morphology, as observed via mutated GFP-ADNP fluorescence, was caused due to tubulin/microtubule reduction. For this purpose, neuron-like cells expressing GFP-ADNP were treated with zinc, which was used as a microtubule-disrupting agent [36]. Increased cytoplasmic/nuclear aberrant morphology was observed following zinc treatment compared to the sham-treated cells (Figure 6A). Importantly, also in this paradigm, NAP treatment corrected the aberrant cytoplasmic/nuclear morphology reflected in indiscernible nuclear–cytoplasmic boundaries (Figure 6B). Similar results were obtained via nocodozole treatment (Figure 6C, images, Figure 6D, quantitation). In contrast, paclitaxel, which stabilized microtubules, did not cause this effect (Figure 6C,D).

### 3.5. No Effect on ADNP Expression

**Figure 7:**
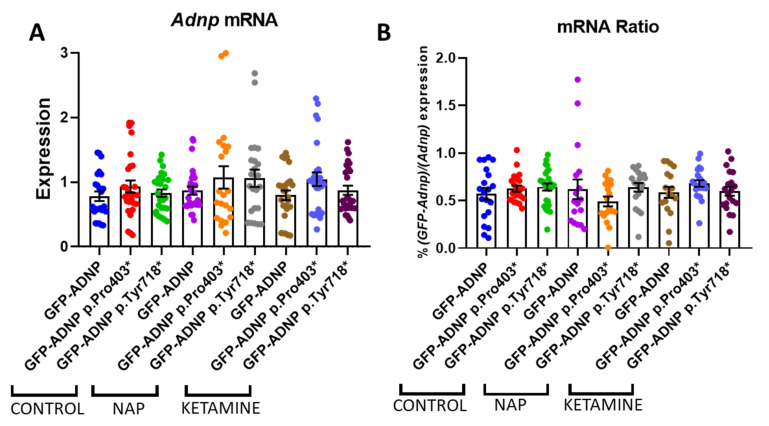
Neither NAP nor ketamine increases ADNP expression. Total RNA was extracted from GFP-ADNP, GFP-ADNP p.Pro403*, and GFP-ADNP p.Tyr718* sham treated or treated with NAP or ketamine. *Adnp* mRNA (**A**) or the relative amount of *GFP-Adnp* mRNA (**B**) was determined utilizing specific primers. Results were normalized to *Hprt*. A two-way ANOVA with Tukey’s post hoc test revealed no significant differences between the treatments.

We further investigated whether NAP treatment, shown before to correct for deficiencies in *Adnp* mRNA expression in a time- and tissue-dependent manner [35], affected ADNP expression levels. Given that excess ADNP will in turn down regulate ADNP expression (autoregulation) [8,59], we used a low but biologically active NAP concentration of 1 pM [3]. Figure 7A shows no the effects of NAP on *Adnp* mRNA transcript level in our experimental settings. We used ketamine as a control, which was described before to increase ADNP content [42]. However, here, ketamine was also ineffective in increasing *Adnp* mRNA (Figure 6A). Figure 7B included the utilization of primers recognizing the *GFP-Adnp* mRNA fusion site, indicating that the *Adnp*-mutated mRNA species amounted to ~0.5% of the total transcripts. Neither NAP nor ketamine treatment affected *Adnp* mRNA levels (Figure 7B).

### 3.6. Ketamine Neither Increases Adnp mRNA Expression nor Protects against Mutation-Associated Aberrant ADNP Nuclear/Cytoplasmic Distribution While Interacting with ADNP at a SIP-Microtubule-Binding Motif

**Figure 8:**
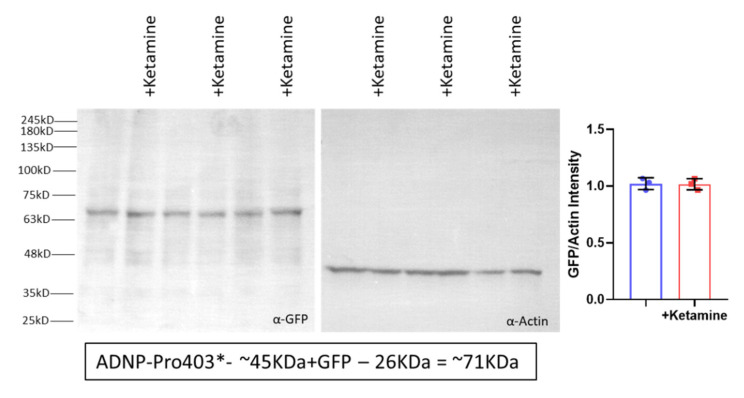
Ketamine does not increase ADNP protein expression in ADNP-mutated neuroblastoma cells. Proteins were extracted from GFP-ADNP p.Pro403* neuronal-like differentiated cells that had been subjected to sham or ketamine (100 µM) treatment for 4 h [33]. Polyacrylamide gel electrophoresis followed by Western blotting was performed as described in Section 2. Molecular weight markers are indicated on the left-hand side [40]. The left panel depicts protein extracts from three independent experiments that were separated and reacted with α-GFP antibodies. As shown in the middle panel, the same blot was reacted with α-Actin antibodies. Blots were subjected to densitometry using ImageJ Fiji software [46]. The graph on the right panel depicts GFP-ADNP p.Pro403* neuronal-like differentiated cells, control, blue bar and GFP-ADNP p.Pro403* neuronal-like differentiated cells + ketamine, red bar, showing identical expression of GFP/Actin.

As indicated above, ketamine was previously suggested to affect ADNP concentrations [42]. As also indicated, here, no effect of low-dose ketamine was observed at the mRNA level (Figure 7). Previous independent publications have suggested that ketamine increases ADNP at the protein level [42]; however, Western analyses in our model systems using the GFP-ADNP p.Pro403* clonal cell line [40] verified the mRNA data (Figure 8).

Additionally, when ketamine was tested to find out whether it had an immediate effect on ADNP-mutant-induced indiscernible nuclear envelopes, our results indicated no effects. Thus, the percentage of diffused GFP-ADNP in the cells was 34.8 ± 2.6 vs. 43.1 ± 3.4 after ketamine addition. Similarly, the diffused percentage of GFP-ADNP p.Pro403* was 36.9 ± 1.9 vs. 42.5 ± 3.7 after ketamine addition, and for GFP-ANDP p.Tyr718*, the value for this metric was 85.5 ± 3.2 vs. 91.7 ± 3.2 after ketamine addition.

**Figure 9:**
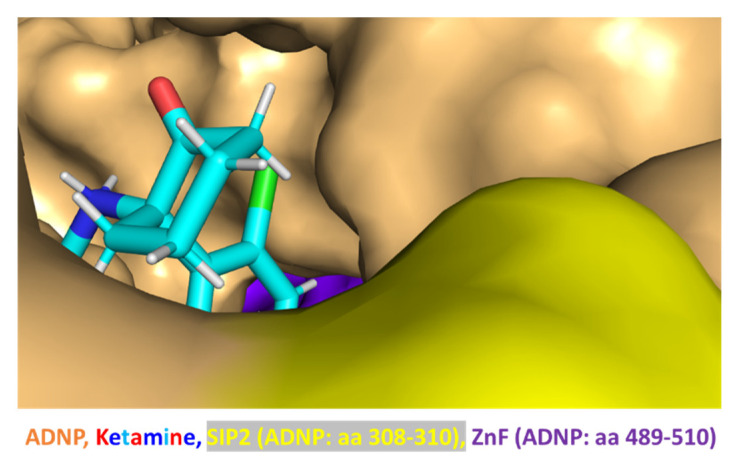
Ketamine interacts with ADNP. Experiments were performed as described in Section 2; the HDOCK and PyMOL software results are shown. ADNP is shown in surface view; ketamine is shown in sticks. Abbreviations include aa = amino acids within the ADNP 1102 aa sequence. SIP2 (single aa code, yellow) is separated from the NAP–NAPV**SIP**Q, aa, 354–361 SIP1 sequence, aa, 358–360 on ADNP (Figure 10). ZnF = zinc finger (purple).

Interestingly, in silico modeling suggested the direct interaction of ketamine and ADNP (Figure 9, close inspection).

**Figure 10:**
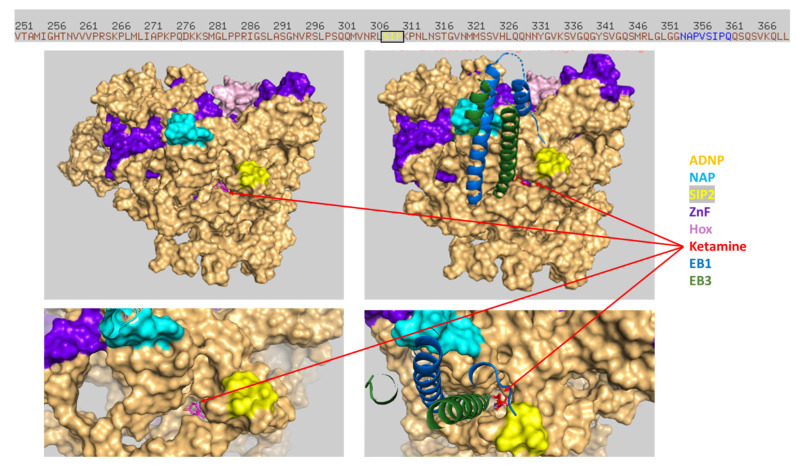
Ketamine interacts with ADNP and EB1/EB3. Experimental means are delineated in Section 2, and the SwissDoc results are shown. The ruler on top includes a partial view of the human ADNP linear amino acid sequence at the site of ketamine association. The NAP motif (NAPVSIPQ, amino acids, 354–361, on ADNP) is indicated in blue (ruler) or cyan (picture), including the EB1/EB3 binding SIP sequence (Figure 9). The second ADNP-SIP motif ADNP amino acids, 308–310 (SIP2), are also shown on the ruler (yellow letters, box). ADNP (surface view). A cartoon representation of EB1/EB3 and ketamine (sticks) is shown, with EB1/EB3 docking being shown on the right-hand side and via a close up at the bottom of the figure. ZnF, zinc fingers; Hox, the ADNP homeobox domain [5,60] (see also Figure 9).

Importantly, the docking of ketamine on the ADNP structure using HDOCK or SwissDock (see Section 2) identified ketamine’s preference for an identical groove within ADNP. The similar docking results, which were obtained using two different software methods, strengthen the finding of the ketamine interaction groove within ADNP. Thus, looking at the entire ADNP sequence (Figure 10, SwissDoc) highlighted the finding that the ketamine docking groove on ADNP included the additional ADNP SIP2 motif (amino acids, 308–310) [4,7,8,9,10,11,12], further binding to microtubule end-binding proteins EB1 and EB3 and constituting a central NAP (NAPV**SIP**Q amino acids 354–361) target [33] (Figure 10, cyan labeled). A surprising additional interaction with EB1/EB3 was also discovered while docking both EB1/EB3 and ketamine on ADNP (Figure 10).

### 3.7. Discovery of NAP Interactions with ADNP Zinc Finger-Binding Motifs

**Figure 11:**
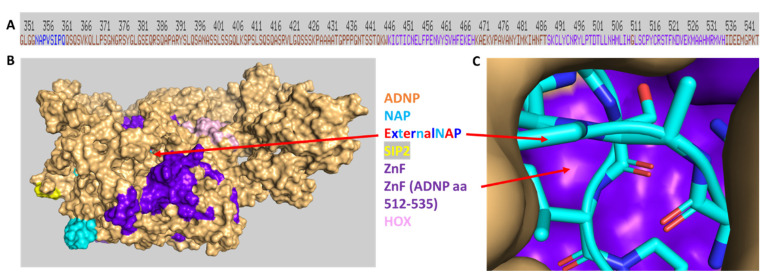
NAP (davunetide) docks on an ADNP zinc finger groove. Experimental means are delineated in Section 2. (**A**) The ruler indicates the human ADNP linear amino acid sequence (single-letter amino acid code). The ADNP internal NAP motif (NAPVSIPQ, amino acids, 354–361, on ADNP) is indicated in blue, including the EB1/EB3 binding SIP sequence. Two ADNP consecutive zinc fingers (ZnF) are indicated in dark purple. (**B**) ADNP, surface view, and external NAP sticks are shown. (**C**) NAP’s association with ADNP in a close-up version. Abbreviations are as in Figure 9 and Figure 10 [5,60].

While we have previously shown NAP (davunetide) interaction with ADNP/EB1/EB3 through its SIP motif [38], here, we aimed to assess external NAP’s most feasible direct association with ADNP. For this purpose, we resorted to HDOCK [54] in silico protein/protein docking. Our results identified a novel NAP interaction site on ADNP (Figure 11A, shown adjacent to two linear zinc finger motifs in dark purple). The three- dimensional overview (Figure 11B) indicates the NAP association site in an ADNP groove linked with zinc finger motifs. Figure 11C, shows direct association of NAP with a zinc finger motif on ADNP (aa 512–535). This zinc finger motif is adjacent to the ketamine-associated zinc finger interaction on ADNP (aa 489–510, Figure 10 and Figure 11A).

## 4. Discussion

Using our recently developed CRISPR/Cas9 ADNP-mutated cell lines [40], we have shown, for the first time, rapid NAP (davunetide) nuclear localization. Furthermore, this rapid nuclear penetrance of NAP was associated with the repair of the previously observed indiscernible nuclear envelope boundaries/ADNP distribution in the face of ADNP mutations. Interestingly, while we revealed significant NAP-enhanced repair, our previous measurements, such as those taken 7 days after NAP addition, showed a more efficacious effect, i.e., complete normalization indicative of a gradual continuous effect. The immediate NAP effects were linked with microtubule protection. Indeed, we have previously shown rapid microtubule reorganization following NAP treatment, as well as the short-term effects of NAP on tubulin expression [61] and microtubule-dependent axonal transport [62]. The long-lasting efficacious effects explain a previous finding showing that a single administration of NAP presents sustainable preventative neuroprotective effects against the deleterious sequela of traumatic head injury in mice [63] that is associated with gene expression regulation [64,65].

Our current results connect the NAP microtubule protection effects with NAP transcriptional activity. Mechanistically, ADNP cytoplasmic localization via 14-3-3 promotes sex-dependent neuronal morphogenesis, cortical connectivity, and calcium signaling [66], and the ADNP/neuroprotective active site NAP protects against cytoskeletal (microtubules/actin) [3], autophagy [67], and calcium signaling-associated pathologies [9], with exogenous NAP enhancing/replacing deficient ADNP. Here, we showed, for the first time, that reduced microtubule content/function led to aberrant ADNP nuclear envelope distribution, and this adverse effect was corrected by NAP, which, in turn, was further localized to the nuclear compartment, potentially interacting with ADNP zinc finger domains.

Our new finding of labeled NAP next to the cytoplasmic membrane (immediately following application) is also of interest and warrants further investigation.

As indicated in the Introduction section, we previously discovered that the cellular targets of NAP (NAPVSIPQ) include the microtubule end-binding proteins 1 and 3 (EB1 and EB3), which are critical to microtubule dynamics and Tau–microtubule interaction [32,33,36]. Furthermore, NAP correlates with SH3 domains and affects SH3-dependent protein interactions, thus playing a part in a variety of physiological and pathological protein–protein associations. For example, impairment in the interaction of the SH3-containing protein SHANK with a wide spectrum of its target cytoskeletal proteins has an impact on autism/schizophrenia pathophysiology, and NAP, through regulating SH3-mediated protein interactions, exhibits a favorable effect on these cytoskeletal interactions and the subsequent cellular processes [3]. Our findings are complemented by the demonstration of NAP interaction with the armadillo motif of beta catenin [16], a transcriptional regulator [68]. Our current discovery of NAP nuclear localization could indicate that it also has a role in the co-regulation of beta catenin transcriptional control. For example, beta catenin has been shown to interact and regulate FOXP2, a highly conserved key player in embryonal development that is important for language acquisition [68]. Our previously published studies have suggested that ADNP/NAP regulate FOXP2 in the tongue [51] and that NAP regulates FOXP2 in the brain [69]. Furthermore, we showed that the expression of ADNP is highly correlated with FOXP2 expression in the brain [70].

ADNP facilitates the robust transcriptional control of hundreds of genes, but this can be disrupted by ADNP deficiency/mutations, affecting multiple organs; however, this can be partially corrected via NAP treatment. This ameliorative NAP effect can be found in the brain [9,35,49], muscles [51], and the immune system [10] and can be corroborated in human tissues and cells [11,12], explaining the complex nature of the neurodevelopmental ADNP syndrome and the breadth of NAP protection in the context of clinical development.

As indicated earlier, NAP treatment can correct for deficiencies in *Adnp* mRNA expression in a time- and tissue-dependent manner [35]. It has been suggested that Low-dose ketamine can protect against ADNP mutations by increasing ADNP expression [42]; however, these findings may be dose- and time-dependent and require further investigation. Furthermore, a recent clinical trial based on this hypothesis did not demonstrate *ADNP* mRNA increases following ketamine application [71]. These results agree with the findings of the present study. Interestingly, high ketamine doses were originally associated with increased apoptosis, prompting the use of NAP as a protective agent [43], with ADNP and NAP (davunetide) directly in involved in protection against apoptosis [72]. The protocol used here included the same incubation period and low concentrations of ketamine, suggested before to increase ADNP [42]; however, based on the current study’s results, ketamine’s direct association with ADNP may invoke additional pathways. Most recently, the same dose of ketamine used here impaired growth cone and synaptogenesis in human GABAergic projection neurons via GSK-3β activation and HDAC6 signaling inhibition [73], with ADNP/NAP possibly protecting against both [34,37,67]. The effects of ketamine may be further mediated through the WNT signaling pathway [74], which is regulated by ADNP/NAP [11,12,16,38]. From an animal model perspective, ketamine administration, like ADNP deficiency (mutations) has been suggested to model autism [75] and schizophrenia [76] in rodents. However, the ketamine dose used in these preclinical studies was higher than the low dose (0.77 mg ketamine/kg/hour), showing the exacerbation of psychotic symptoms and cognitive impairment in neuroleptic-free schizophrenics [77]. Notably, in a double-blind clinical trial, ketamine was ineffective in adolescent children suffering from autism [78]. Our novel results suggest that part of the ketamine effects may be a consequence of ADNP binding changing ADNP/NAP/EB1/EB3 interactions.

In summary, for the first time in the literature, the present study showed that rapid NAP (davunetide) activity repairs ADNP distribution, and this is mediated by microtubule protection and nuclear localization. Our in silico modeling suggested external NAP (davunetide) interactions with an ADNP zinc finger groove, potentially alluding to their influence on DNA transcription prior to correcting ADNP deficiencies [9,10,35,49,51]. These findings align with brain bioavailability in animal models and humans, as well as safe toxicology profiles and positive clinical experiences in adults [79,80]. Further, the US Food and Drug Administration’s (FDA) orphan drug designation and rare pediatric disease designation make davunetide an ideal first-choice drug candidate for further clinical development in the context of ADNP syndrome and beyond [79].

## 5. Conclusions

NAP (davunetide) partly ameliorates transcriptional deficiencies associated with ADNP mutations. This transcriptional control may now be explained by the newly discovered nuclear localization, which is mediated by microtubule protection and associated with nuclear ADNP/NAP interactions.

## 6. Patents

NAP (CP201, davunetide) use is under patent protection (US patent nos. US7960334, US8618043, and USWO2017130190A1) (I.G.), PCT/IL2020/051010 (I.G.), and PCT applications (I.G., inventor and Y.I.-P., M.G., and G.K., contributing scientists).

## Data Availability

Not applicable.
